# Hybrid-Based Wound Dressings: Combination of Synthetic and Biopolymers

**DOI:** 10.3390/polym14183806

**Published:** 2022-09-12

**Authors:** Blessing Atim Aderibigbe

**Affiliations:** Department of Chemistry, University of Fort Hare, Alice 5700, Eastern Cape, South Africa; baderibigbe@ufh.ac.za

**Keywords:** hybrid wound dressings, synthetic polymers, skin regeneration, natural polymers, wound healing, wounds, wound dressings, biomaterials

## Abstract

Most commercialized wound dressings are polymer-based. Synthetic and natural polymers have been utilized widely for the development of wound dressings. However, the use of natural polymers is limited by their poor mechanical properties, resulting in their combination with synthetic polymers and other materials to enhance their mechanical properties. Natural polymers are mostly affordable, biocompatible, and biodegradable with promising antimicrobial activity. They have been further tailored into unique hybrid wound dressings when combined with synthetic polymers and selected biomaterials. Some important features required in an ideal wound dressing include the capability to prevent bacteria invasion, reduce odor, absorb exudates, be comfortable, facilitate easy application and removal as well as frequent changing, prevent further skin tear and irritation when applied or removed, and provide a moist environment and soothing effect, be permeable to gases, etc. The efficacy of polymers in the design of wound dressings cannot be overemphasized. This review article reports the efficacy of wound dressings prepared from a combination of synthetic and natural polymers.

## 1. Introduction

Wounds are challenging to treat, especially when they have been invaded by microbes [1,2]. Wound healing involves complex mechanisms that require using appropriate wound dressings to induce a timely wound healing process [3]. Polymers have played a huge role in the design of potent wound dressings. Synthetic and natural polymers are used to prepare wound dressings. Synthetic polymers are characterized by features such as easy preparation resulting in controlled physicochemical properties and stability, good mechanical stability with interesting mechanical properties, and degradation in a controlled manner [4,5]. However, they can induce the risk of toxicity and are biologically inert. Natural polymers, on the other hand, do not offer interesting mechanical properties, are biocompatible, biodegradable with interesting biological activities, and can undergo enzymatic degradation to produce by-products that do not trigger toxic reactions [6,7]. However, their high rate of degradation rates is usually challenging to control [5].

The combination of synthetic polymers together with natural polymers has been widely employed to overcome shortcomings common with both types of polymers [8,9]. The combination of both polymers in the design of wound dressings results in hybrid-based wound dressings. Hybrid wound dressings display excellent features such as improved mechanical properties, accelerate wound healing, excellent flexibility, biocompatibility, biodegradability, high adsorption capacity, etc. [10,11]. They are also appropriate for treating high exuding, bleeding, and infected wounds. They are also suitable to promote skin regeneration [12,13]. The further incorporation of bioactive agents and biological molecules into hybrid-based wound dressing has resulted in materials that exhibit excellent wound healing and skin regeneration [14,15]. This review reports the in vitro and in vivo outcomes of hybrid-based wound dressings.

## 2. Wound Healing Mechanisms

**Figure 1:**
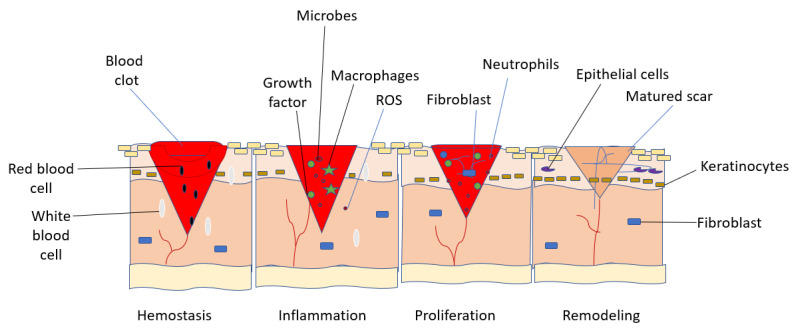
The phases of wound healing.

The physiological and architectural restoration of the skin after an injury is based on four important phases: hemostasis, inflammation, proliferation, and remodeling (Figure 1) [16,17]. Wound healing is complex and specialized cells are involved, such as macrophages, fibroblasts, platelets, endothelial cells, etc. There is a significant interaction between the cells and the extracellular matrix. Wound healing is also influenced by the action of growth factors, chemokines, cytokines, chemokines, receptors, etc. [18,19].

Hemostasis

This phase occurs immediately after an injury, whereby the damaged blood vessels constrict rapidly with a blood clot formation to prevent excess loss of blood [20,21]. The platelets play a crucial role in blood clot formation [22]. The constriction of the blood vessels together with the formation of a blood clot results in the lack of oxygen, changes in pH, and glycolysis [23]. After the narrowing of the blood vessels, the widening promotes the invasion of the wound matrix by thrombocytes. The widening of the blood vessels known as vasodilation is characterized by oedema and local redness of the wound [23]. The blood clot is composed of cytokines, fibrin molecules, growth factors, vitronectin, thrombospondins, and fibronectin. The blood clot also acts as a shield against bacteria invasion and as a reservoir for cytokines and growth factors for wound repair [24]. After the formation of a blood clot, the coagulation process terminates to prevent excessive thrombosis and platelet aggregation [24]. In this phase, the repair of smooth muscle cells and endothelial cells also occurs due to the release of platelet-derived growth factors [25]. The platelet-derived growth factors recruit neutrophils and monocytes, which together with transforming growth factor β (TGF-β) from the vasculature induce the inflammatory response [26]. Fibroblasts are also recruited by platelet-derived growth factors, and they migrate to the wound site followed by the production of collagen, glycosaminoglycans, and proteins which promote cellular migration and set the stage for subsequent healing events [23,24].

Inflammation

The inflammation phase overlaps with the hemostasis phase. In this phase, neutrophils and monocyte infiltration in the wound bed prevent the invasion of microbes, foreign debris, and tissue damage [27,28]. The neutrophils promote the process of phagocytosis of debris and microbes to allow decontamination of the wound. The three known mechanisms of neutrophils in the destruction of debris and bacteria are: directly ingesting followed by destroying the foreign debris by a process known as phagocytosis; via the release of toxic substances, e.g., lactoferrin, cathepsin, proteases, etc. to destroy bacteria and the dead host tissue; and by the production of chromatin and protease ‘traps’ to capture and destroy bacteria in the extracellular space [29,30]. The by-product of the neutrophil activity, oxygen-free radicals, exhibit bacteriocidal properties to sterilize the wound. However, the oxygen-free radicals can exasperate inflammation of the microenvironment, thereby resulting in delayed wound healing [29,31].

After the completion of the neutrophil activity, the neutrophils undergo apoptosis and are phagocytosed by macrophages or can be sloughed from the wound surface [30]. Macrophages are attracted to the wound and are released from platelets and damaged cells. In this phase, they can withstand the acidic wound environment [32]. Macrophages are composed of significant amounts of growth factors, which include TGF-β and epidermal growth factors, which are crucial in regulating the inflammatory response [30,33,34]. The inflammatory phase of wound healing can be prolonged to remove excess bacteria and debris from the wound. Prolonged inflammation can result in significant tissue damage and delayed proliferation, resulting in the formation of a chronic wound [30]. Multiple factors have been reported to affect the immune response such as lipoxins and the products of arachidonic acid metabolism, thereby hindering the next phase of wound healing [35]. Furthermore, the type of immune response plays a crucial role in the formation of hypertrophic scarring or keloid formation. The differentiation of T-helper (Th) cells, major immune mediators in the inflammatory phase to Th2 cell types, results in hypertrophic scarring [36]. In chronic non-healing wounds, the presence of an abundance of neutrophils makes them become proteolytic environments composed of host-derived proteases. Excessive tissue damage is caused by elevated inflammatory cytokines and collagenases [37].

Proliferation

In this phase, there is a complex simultaneous combination of fibroblast migration, angiogenesis, and epithelialization together with a wound retraction [38]. A sufficient supply of gas, blood, nutrients, and metabolites is required [38]. The release of vascular endothelial growth factor (VEGF) and cytokines induces the endothelial cells to promote angiogenesis, the formation of new blood vessels and the repair of damaged blood vessels in the wound site. The migration of fibroblast results in the production of fibronectin and collagen with a replacement of the clot with granulation tissue made up of different ranges of collagen [29]. The fibroblasts are converted to myofibroblast phenotype, which is useful in wound contraction. The myofibroblasts also induce angiogenesis, and the collagens produced by the fibroblasts are responsible for providing strength to the tissues. However, the formation of a hypertrophic scar can be induced by an overproduction of collagen [39]. Several macrophage-derived molecules, such as IL-1, β-bb (PDGF-bb), IL-6, etc. promote pro-re-epithelialization molecules in the fibroblasts. In wounds without a macrophage-derived molecule, IL-6, there is a lack of appropriate inflammatory response, resulting in hampered collagen accumulation, angiogenesis, and re-epithelialization [40]. The migration of the epithelial cells from the edges of the wound forms a sheet of cells that covers the wound, and this process is known as epithelialization [41]. Epithelialization takes place within 24 h in primary wounds, but in secondary closed wounds, the contraction of large areas lacking epithelial cells occurs before complete epithelialization [29,30].

Remodeling

This last phase is characterized by a transition from granulation tissue to scar formation with slow angiogenesis and a replacement of type III collagen with type I collagen that is stronger [28]. This remodeling phase is significantly promoted by myofibroblasts developed from fibroblasts, which are responsible for wound contraction [42]. Some reports have shown that fat cells obtained from the differentiation of myofibroblasts replenish the subcutaneous adipose tissue, and this is influenced by the neogenic hair follicles, resulting in the activation of adipocyte transcription factors and bone morphogenic protein (BMP) signaling [43].

### Chronic Wounds

Chronic wounds are classified as non-healing wounds over a prolonged period, and they can be classified as diabetic, vascular, diabetic, and pressure wounds [44]. Other examples of chronic wounds are gangrenes, ischemia, etc. [45]. The most common type of chronic wound is the diabetic wound, and it affects 15% of the world population who suffer from diabetes [46]. Due to the prolonged healing period of these types of wounds, the patients usually require long medical care, amputation, and in some cases, long hospital stay [47]. Factors that contribute to chronic wounds are reduced blood supply and re-epithelialization, inflammatory responses that are not controllable, and bacterial infections.

There are complications associated with diabetes such as foot infections, etc. Uncontrolled diabetes reduces the tissue oxygen rate, thereby damaging blood vessels and forming non-healing ulcers [48]. Venous ulcers are common in older patients and affect the lower limbs due to a damaged deep venous system. It is also characterized by increased blood pressure in the vessels, causing leakage and an accumulation of fibrin that blocks the vascular pathway, reducing the flow of oxygen to the surrounding tissues [45,48]. Pressure ulcers are also common in older and paralyzed patients. It occurs due to continuous pressure on the skin, decreasing the diffusion of oxygen in the tissues [45].

#### Pathophysiology of Chronic Wounds

**Figure 2:**
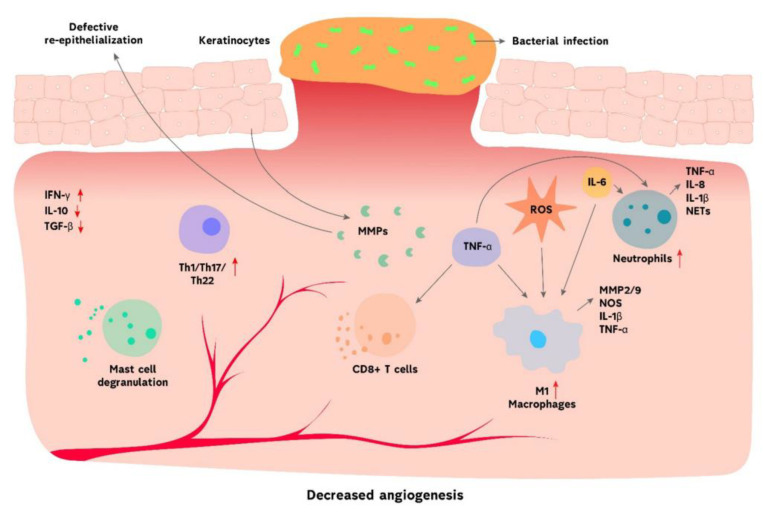
The immune environment of chronic wounds [50].

Chronic wounds remain in the inflammatory stage of wound healing due to the persistent recruitment of neutrophils and macrophages in the wound bed, which also prolongs the wound healing process (Figure 2) [45,49]. The high production of inflammatory molecules and Reactive Oxygen Species (ROS) in chronic wounds affect the synthesis of collagen, decreases proliferation, and causes an abnormal differentiation of keratinocytes [49]. The altered pattern of cytokine in the wound also contributes to delayed wound healing in diabetes patients. The abnormal expression of growth factors is observed in diabetic foot ulcers that disrupt the healing process. Elevated levels of matrix metalloproteinases and reduced levels of tissue inhibitors of metalloproteinases in chronic wounds also affect the wound healing process [45,49]. Matrix metalloproteinases are crucial for the remodeling of the matrix microenvironment by inducing healing responses, such as cellular migration, proliferation, and angiogenic induction. The high levels of protease results in the damage of extracellular matrix (ECM) and growth factors degradation together with their receptors [50]. The damaged ECM prohibits the wound healing process from moving to the proliferative phase. The aforementioned factor results in an inflammation cycle where more proteases are produced. The persistent inflammatory and hypoxic state of chronic wounds induce a high production of ROS that also destroys the ECM proteins.

The presence of senescent macrophages, keratinocytes, fibroblasts, and endothelial cells in chronic wounds contributes to oxidative stress that results in the damage of deoxyribonucleic acid (DNA) cell cycle arrest and a defect in the intracellular biochemical pathways including GSK-3β/Fyn/Nrf2 pathway, etc. [49,50]. Chronic wound healing is characterized by reduced angiogenesis and tissue epithelialization [50]. The mesenchymal stem cells also play an important role in wound healing and are recruited into circulation when there is an injury. However, they are defective and deficient in chronic wounds. The absence of effective receptors or promigratory matrix substrates inhibits cell migration and proliferation in chronic wounds. The hallmarks of chronic wounds have been reported to be impaired neovascularization and angiogenesis, resulting in an insufficient supply of nutrients and oxygen for the cells in the wound bed, leading to non-healing wounds [49]. To accelerate the wound healing process of chronic wounds, researchers have employed the use of microbial agents to treat persistent microbial infections [51,52], the delivery of healthy donor-derived functional mesenchymal stem cells to deal with their deficiency [53], and the administration of antioxidants to reduce the ROS to normal levels, thereby reversing the chronic state of the wounds [54,55].

In the treatment of chronic wounds, debridement is performed to remove non-viable tissues [56]. Anti-inflammatory agents are also employed to deal with prolonged inflammation [57], and the use of appropriate wound dressings that addresses the moisture imbalance is crucial for treating chronic wounds [58]. To promote the formation of granulation tissue and epithelialization, the use of growth factors has been used [59,60].

## 3. Hybrid Wound Dressings in Wound Healing

Hybrid-based wound dressings have been developed from the combination of natural and synthetic polymers. Different types of hybrid-based wound dressings have been developed, such as foams, hydrocolloids, hydrogels, nanofibers, films/membranes, etc. Different preparation techniques have been employed. This section reports the preparation techniques used as well as in vitro and in vivo biological outcomes.

### 3.1. Foams

**Figure 3:**
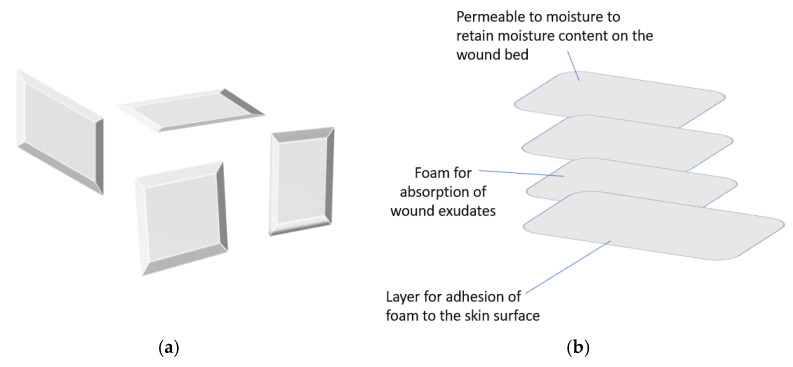
(**a**) Foam-based wound dressings, (**b**) Multi-layer foam.

The commonly used foam wound dressings are polyurethane foams used for moist wound healing (Figure 3a,b) [61]. Silicone foams are not commonly used. Foam wound dressings are characterized by a porous structure and a film-backing, and they are produced with varied thicknesses [62]. They can be adhesive or non-adhesive. The permeability of the film backings varies with a significant influence on water evaporation and gas exchange capacity [62]. The contact of the wound with the foam products is crucial to facilitate the absorption of the exudate. Their adhesion to the surrounding skin of the wound bed is useful in keeping the dressing and preventing the leakage of the exudates that can cause skin irritation [63]. They are suitable as primary or secondary wound dressings. Some of their unique features include their ability to maintain moisture at the wound bed, to be easily removed from the wound, to protect the wound from bacterial invasion, to provide mechanical protection, and to conform to the body shape; they are also non-toxic, easy to use, etc. [62]. Foams are effective for the management of acute and chronic with medium to heavy exudate [64]. They have been prepared from a combination of synthetic and natural polymers.

Most reported foam wound dressings are prepared from synthetic polymers [65,66]. Some are prepared from natural polymers only with high porosity but are prone to enzymatic degradation [67,68]. Hybrid-based foams have been prepared from polyurethane in combination with natural polymers, such as chitosan, sodium alginate, and hydroxypropyl methylcellulose, by Namviriyachote et al. The foams were loaded with silver nanoparticles and asiaticoside. Foams prepared from alginate displayed a high release of silver nanoparticles and asiaticoside. Using natural polymers for the preparation of the foams influenced the compressive strength and absorption properties. The foams were non-cytotoxic and compatible, and they improved the rate of wound closure with a significant formation of granulation tissue [69]. A multi-layer wound dressing comprising foam was developed from polyvinyl alcohol with a surface mesh designed from sodium carboxymethylcellulose. The wound dressing was loaded with stearyl trimethyl ammonium chloride, which is an antimicrobial drug into the foam layer for the prevention and control of infection prepared by He et al. The surface mesh was designed for hemostatic function. The foam layer displayed excellent absorption capacity of excess exudate with a good inhibition effect against *E. coli* and *S. aureus* with good hemostatic capability [70].

### 3.2. Hydrocolloids

**Figure 4:**
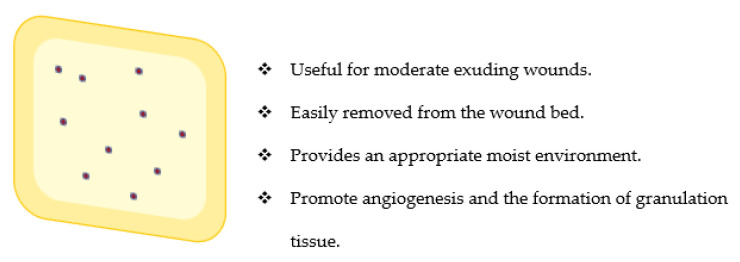
Hydrocolloid wound dressings.

Hydrocolloid dressings are made up of an outer layer that is water-impermeable and an inner layer of gel-forming material commonly prepared from biopolymers, such as pectin, sodium carboxymethylcellulose, gelatin, etc. (Figure 4) [71,72]. They are useful for moderate exuding wounds which include burn wounds, pressure sores, etc. [71,73]. They are easily removed without causing pain. When they come in contact with the wound exudates, gels are formed, which provide an appropriate moist environment with good absorption of the wound exudates [71,72,73]. They promote angiogenesis, induce the formation of granulation tissue, and increase the number of dermal fibroblasts and the amount of synthesized collagen [72]. Their capability to retain moisture is useful in softening and rehydrating necrotic tissue and slough, thereby promoting autolytic debridement [72]. However, they are not effective in the management of high-exuding wounds [74].

The gel formation when in contact with exudates makes hydrocolloid dressing easy to remove, and also, reduces pain in the wound bed by protecting the nerve endings [72,74]. It is cost-effective when compared to traditional wound dressings, such as gauze when used for the treatment of pressure ulcers [75]. The frequency at which it is changed is 3–7 days depending on the amount of exudate produced [72,76]. They are designed to be intact when in contact with the wound without a breakdown into the wound and also prevent scarring. They have been developed from a combination of biopolymers and synthetic polymers.

Hydrocolloids have been prepared from a combination of sodium carboxymethyl cellulose, styrene–isoprene–styrene, and silk fibroin nanoparticles by Lee et al. [77]. The addition of silk fibroin nanoparticles improved the water uptake, swelling ratio, and tensile strength of the hydrocolloids. The hydrocolloids were non-toxic, improved the structural integrity of wound tissue, supported the regeneration of the dermis layer, decreased the burn wound size, and increased the density of collagen fibres in vivo. These findings revealed the efficacy of combining a biopolymer with synthetic polymers for the design of hydrocolloids [77]. Jin et al. prepared hydrocolloid wound dressings from a combination of sodium alginate, styrene–isoprene–styrene copolymer and petroleum hydrocarbon resin using the hot melting method [78]. The hydrocolloids were loaded with *Centella Asiatica*. The hydrocolloids displayed excellent mechanical properties, and they provided a *moist* environment for accelerated epithelialization and wound healing. The presence of sodium alginate in the wound dressing played a crucial role in the accelerated rate of wound healing in vivo. Hydrocolloids have also been prepared from a combination of styrene–isoprene–styrene and carboxymethyl cellulose sodium salt followed by loading myrrh solution. The tensile strength of the hydrocolloid was significant (1180.92 ± 84.05 kPa) when compared to the commercial hydrocolloid (621.74 ± 96.52 kPa). The swelling capability of myrrh plays a vital role in the mucoadhesive property, mechanical properties, and water uptake of the fabricated hydrocolloids. They were biocompatible and induced accelerated collagen deposition, and regeneration of dermis, mature epidermis, and hair follicles in vivo [78]. The combination of biopolymer with synthetic polymers for the design of hydrocolloids resulted in biocompatible wound dressings that promoted accelerated wound healing. However, there are very few studies on the effect of biopolymers in hydrocolloid-based wound dressings.

### 3.3. Hydrogels

**Figure 5:**
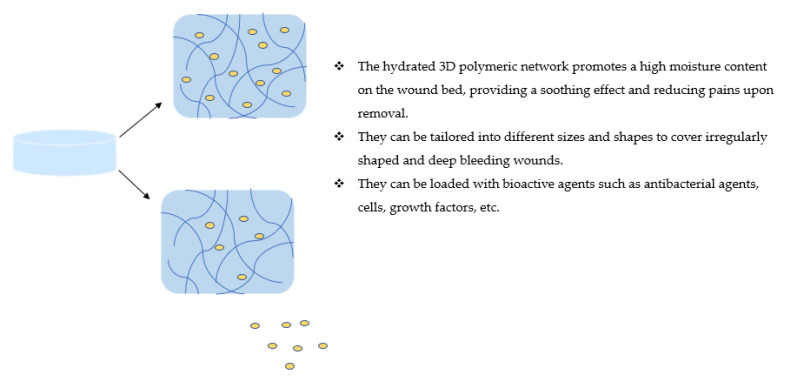
Hydrogels loaded with bioactive agents.

Hydrogels represent a class of materials that are three-dimensional (3D) networks composed of crosslinked polymers (Figure 5) [79,80]. They are insoluble and demonstrate a remarkable potential to absorb aqueous media, making them suitable for wound dressings. They are porous and allow oxygen diffusion appropriate for accelerated wound healing [81,82]. Their hydrated 3D polymeric network promotes a high moisture content on the wound bed by providing a soothing effect and reducing pains upon removal [83]. Their hydrophilic properties result from the crosslinking density of selected polar functional groups, such as hydroxyl, amide, carboxyl, amino, etc. [84]. They can be tailored into different sizes and shapes to cover irregularly shaped and deep bleeding wounds [85]. They can be loaded with bioactive agents, such as antibacterial agents, cells, growth factors, etc. [86,87]. They resemble the extracellular matrix by providing a compatible environment for cellular proliferation and tissue regeneration, making them biocompatible and appropriate for the management of wounds [88]. Hydrogels have been formulated to display a stimuli-responsive manner for controlling drug release mechanisms, making them interactive dressings [89]. However, their application as wound dressings is limited by poor mechanical properties, making them unsuitable for the treatment of excessive bleeding and the prevention of secondary damage [90]. Many researchers are currently developing hydrogel dressings with high mechanical strength by combining synthetic and biopolymers.

Kong et al. developed poly (vinyl alcohol)/sodium alginate hydrogels loaded with 5-hydroxymethylfurfural, an antioxidant and silver nanoparticles [91]. Loading 5-hydroxymethylfurfural effectively induced the proliferation and migration of human skin fibroblasts and collagen production with a controlled release. The hydrogel displayed good compatibility and accelerated wound healing in vivo by increasing collagen production, enhancing angiogenesis/vascularization, inducing re-epithelialization, and improving inflammation. Increasing the content of sodium alginate in the hydrogel promoted a porous structure when compared to the polyvinyl alcohol hydrogels that displayed a densely packed network. The presence of sodium alginate also influenced the swelling capability of the hydrogel network. The hydrogel wound dressings’ water retention capacity made it possible for the hydrogels not to cause a secondary injury upon removal from the wounds. The hydrogels absorbed exudates from the wounds and also provided a moist environment for the wounds. The hydrogels displayed controlled drug release, suppressed bacteria growth, and modulated the inflammatory response [91].

**Figure 6:**
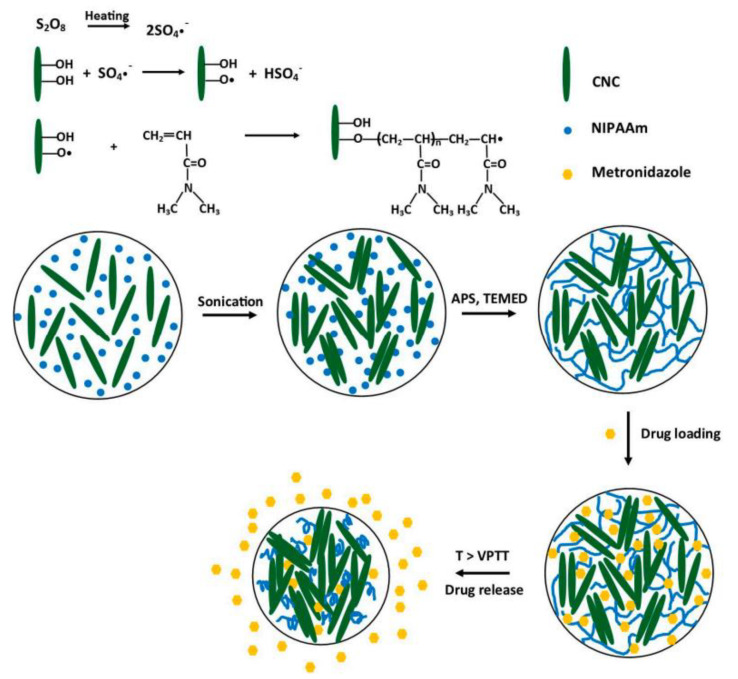
Synthesis of poly(*N*-isopropyl acrylamide)-nanocellulose hydrogels [92].

Zubik et al. designed thermo-responsive hydrogels from a combination of poly(*N*-isopropyl acrylamide) and cellulose nanocrystals via free-radical polymerization without the use of additional crosslinkers (Figure 6) [92]. Increasing the content of cellulose crystals decreased the thermal stability of the hydrogels. The elastic and viscous moduli of hydrogels were improved by the amounts of cellulose crystals, revealing the enhanced mechanical properties of the hydrogels. The thermo-responsive behaviour of the hydrogels was evident between 36 and 39 °C, which is a physiological temperature. Loading the hydrogel with metronidazole, an antibiotic, revealed a slow and sustained drug release at 37 °C [92].

Rasool et al. developed hydrogels from the combination of chitosan, poly (*N*-vinyl-2-pyrrolidone), and poly acrylic acid (PAA) by the solvent-casting technique [93]. The hydrogels were thermally stable with good antibacterial activity against *E. coli* and biodegradable. The swelling behaviour of the hydrogels was influenced by the content of poly (*N*-vinyl-2-pyrrolidone). Increasing the content of poly (*N*-vinyl-2-pyrrolidone) decreased the swelling capacity of the hydrogels. The hydrogel was loaded with silver sulfadiazine, which is an antibiotic that displayed a 91.2% drug release in 1 h in a controlled manner [93]. Xue et al. designed a quaternized chitosan–Matrigel–polyacrylamide hydrogel [94]. The hydrogel displayed a high swelling ratio, excellent mechanical properties which include good adhesiveness, excellent compressive and stretchable properties, and a modulus of 24.6 kPa similar to human skin. In vivo studies showed that the hydrogel promoted collagen deposition, induced skin adnexal regeneration, and accelerated wound healing. The addition of Matrigel enhanced the pore sizes of the hydrogels. The porous nature is useful for maintaining a moist environment for cellular proliferation and the formation of extracellular matrix-like structures for accelerated wound healing. The mild adhesive capacity of 1.3 kPa of the hydrogel on porcine skin indicated their capability to prevent secondary damage upon removal from the wound and their capability to protect the wound from infection. The incorporation of quaternized chitosan contributed to the antimicrobial activity of the hydrogel. The hydrogels also displayed hemostatic, antimicrobial and adhesive properties useful for full-thickness skin defects [94]. Khorasani et al. reported polyvinyl (alcohol)/chitosan/nano zinc oxide hydrogels prepared by a freeze–thawing method [95]. Increasing the number of freeze–thawing cycles increased the hydrogel porosity appropriate for the absorption of wound exudates. Increased elastic modulus and tensile strength, with a decreased elongation at the breakpoint, were observed due to the increased number of freeze–thawing cycles. The antibacterial properties of the hydrogels against *E. coli* and *S. aureus* increased, revealing the synergetic effect of nano zinc oxide (nZnO) with chitosan. The hydrogels were non-toxic, biocompatible, and potential wound dressings for treating infected and exuding wounds [95]. Chen et al. encapsulated vascular endothelial growth factor into benzaldehyde-terminated polyethylene glycol and dodecylmodified chitosan hybrid hydrogel [96]. The hydrogel was prepared using a reversible Schiff base. The hydrogel displayed excellent tissue adhesion, hemostasis, and blood cell coagulation effects. The loaded bioactive agent promoted cell proliferation together with tissue remodeling and reduced inflammatory response suitable for promoting wound healing. An in vivo study revealed the hydrogel’s capability to repair acute tissue injuries including vessel and liver bleeding due to its excellent adhesion and hemostasis features. The dodecyl groups in the hydrogel network exhibit a significant binding affinity to the cytomembranes on the tissue surface. The hydrogels are integrated into the irregular wound bed, providing an environment for the promotion of cell proliferation and the formation of new epidermis This hydrogel is a good multifunctional hydrogel for infectious and bleeding full-thickness skin defects [96].

Masood et al. loaded silver nanoparticles into chitosan–poly ethylene glycol hydrogel to accelerate the healing of diabetic wounds [97]. The crosslinking of the hydrogel was performed using glutaraldehyde. The hydrogel displayed a high porosity, swelling capability and water vapour transition rate. The porous network accelerated the rate of healing, resulting from a high oxygen penetration to the wound bed and high absorption of exudates, and it provided a suitable microenvironment for enhanced cell attachment. The hydrogel antimicrobial and antioxidant properties were significant in vitro with improved wound healing in vivo in diabetic rabbit models. The zone of inhibition of the hydrogel against *B. subtilis*, *E. coli*, *S. aureus*, and *P. aeruginosa* was 15.5 ± 0.8, 20.2 ± 1.0, 21.5 ± 0.5, and 21.8 ± 1.5 mm. The release of the nanoparticles was sustained for 7 days with slow biodegradation [97].

Jangde et al. prepared a multiphase hydrogel loaded with quercetin-loaded liposomes [98]. The hydrogel was prepared using 15% carbopol and different ratios of gelatin ratio. In vivo studies on albino rats revealed accelerated wound healing with a significant wound closure time. The hydrogel displayed a good water vapour transmission rate of 1477.64 g/m^2^/24 h and a swelling ratio of 3.24 ± 0.23. Increasing the content of gelatin increased the water vapour transmission rate, hemocompatibility and swelling ratio [98]. Xuan et al. reported injectable self-healing hydrogels prepared from a combination of carboxymethyl chitosan and aldehyde functionalized sodium alginate through a Schiff-based reaction [99]. Nanofibers were embedded in the prepared hydrogels, and they had no significant influence on the self-healing capability of the hydrogels. The hydrogels displayed excellent self-healing capability and were easily injectable. The hydrogel promoted wound healing in vivo in a full-thickness wound model. The hydrogels facilitated re-epithelialization together with vascularization and skin regeneration. The mechanical properties were improved due to the dual crosslinking. The hydrogels have potential application for the repair of full-thickness skin wounds [99]. Li et al. developed self-healing hydrogel, poly-(ethylene glycol)–chitosan via the Schiff base. Its ability to change shape with fluidlike mobility made it an excellent drug carrier, which was adapted to the internal tissue environment, covered irregularly shaped wounds, and displayed a good hemostatic effect in vivo [100]. Kaur et al. designed poly(vinyl)alcohol–sodium alginate-based hydrogels crosslinked using boric acid and calcium ions to overcome antibiotic-resistant pathogens, which is a challenge in the treatment of infected burn wounds [101]. The content of sodium alginate in the polymer network influenced the gel fraction by increasing it significantly. The maximum tensile strength of hydrogel decreased when the content of sodium alginate increased. However, Young’s modulus and elongation at break were improved. The hydrogel was hemocompatible, non-hemolytic and displayed good self-adherence, high swelling capability and antibacterial effect. In vivo studies on Methicillin-resistant *S. aureus* (MRSA)-induced murine burn wound model revealed the efficacy of minocycline-coated hydrogel against drug-resistant bacterial infection [101]. Yin et al. prepared hydrogels using arginine-based poly(ester urea urethane) and glycidyl methacrylate-modified chitosan. The hydrogels displayed a microporous network structure with high water absorption ability and biodegradation with no cytotoxic effect and good antibacterial activity. They exhibited an excellent antibacterial effect by eliminating 91.81% of *E. coli* and 85.59% of *S. aureus* [102]. 

A hybrid hydrogel designed for diabetic wounds was reported by Li et al. [103]. The hydrogel was prepared from a combination of methacrylic anhydride, hyaluronic acid, and polyhexamethylene biguanide. The hydrogel was loaded with sodium alginate–salidroside microspheres for the sustained release of salidroside, which is useful to induce angiogenesis. The hydrogel was prepared by photocrosslinking and was effective against *E. coli* (97.85%) and *S. aureus* (98.56%). In vivo studies using diabetic rat models revealed that the hydrogel promoted the high formation of granulation tissue together with the formation of subcutaneous capillary and high collagen deposition when compared to the blank control, suggesting accelerated wound healing. Polyhexamethylene biguanide was loaded into the hydrogel to induce antibacterial activity. Salidroside is known to exhibit anti-inflammatory, angiogenic, and antioxidant activities. The combination of an antibacterial agent together with an angiogenic agent in the hydrogel accelerated the healing of diabetic wounds [103]. There is a need for more exploration of the combination of antibacterial and angiogenic agents in wound dressings for the treatment of chronic wounds. Hyaluronic acid has also been explored for the design of hydrogels for wound healing. Arginine-poly(ester amide) was photocrosslinked with hyaluronic acid to form hybrid hydrogels. An increase in the content of arginine–poly(ester amide) in the hydrogels improved the physicochemical properties of the hydrogels significantly. The hydrogels induced keratinocytes and accelerated re-epithelialization. Re-epithelialization of the wound bed was significant on the eighth day after post-surgery with a high density of the new tissue formation, resulting from the collagen deposition of fibroblasts. On day 12 of the post-surgery, a matured thick layer of the new epidermis around the wound was visible with a uniform density of collagen deposition [104].

Hybrid hydrogels composed of poly(vinyl alcohol) and hydroxypropyl cellulose were prepared by successive freezing/thawing. Varied amounts of bovine serum albumin were added together with reduced glutathione. The hydrogels were characterized by an interconnected porous structure with an average pore size in the range of 20.7–26.7 μm. The excellent swelling degree and hydrophilicity of the hydrogel were noted. The stability and porosity of the hydrogels were influenced by the content of bovine serum albumin. The release of neomycin trisulfate from the hydrogels was controlled and also suitable for treating infected wounds. Glutathione in the hydrogels played a crucial role in the wound healing capability of the hydrogels by retaining the functional integrity of the hydrogel [105]. Hybrid-based hydrogels were prepared from a combination of polyvinyl alcohol and sodium alginate followed by the incorporation of microspheres designed from polycaprolactone loaded with basic fibroblast growth factor (bFGF) for an accelerated wound healing process. The hydrogel was developed via the freeze–thawing method, and the microspheres were prepared by a double-emulsion solvent evaporation technique. An increase in the content of sodium alginate influenced the porosity, degradation, swelling ability, and elasticity of the hydrogels. However, the increased content of sodium alginate in the hydrogel reduced the elongation at break and maximum strength. The loaded microspheres in the hydrogel provided a sustained release of bFGF but reduced the mechanical strength of the hydrogels. In vivo studies on a burn-wound rat model revealed accelerated wound closure, cell-induced tissue regeneration and wound healing. The hydrogels were also effective against *S. aureus* and *E. coli* growth [106].

Tajik et al. fabricated hybrid hydrogels from a combination of polyvinyl pyrrolidone and fibrous protein keratin via UV irradiation. The hydrogel composed of 3:1 (polyvinyl pyrrolidone: keratin) induced antibacterial properties against both Gram-negative and Gram-positive bacteria, confirming their potential applications in wound dressing. The swelling and tensile strength of the hydrogel were the highest compared to other hydrogels. The release of lavender loaded in the hydrogel was a two-phase profile, an initial burst release from the swollen hydrogel, and a second phase resulting from the extract diffusion and hydrogel degradation [107]. Lavender oil has antibacterial and antifungal effects and is useful for wound healing, promoting the accelerated formation of granulation tissue, collagen synthesis and inducing the differentiation of fibroblasts [108,109]. A multifunctional hybrid hydrogel prepared from a combination of polyvinyl alcohol, sodium alginate, and dopamine was loaded with bis-quaternary triphenyl-phosphonium salt (BTPP^+^). The hydrogels were prepared by a freeze–thawing method and displayed prolonged antimicrobial properties [110]. The addition of dopamine enhanced the antibacterial effect of the hydrogel. The combination of dopamine and BTPP^+^ promoted the aggregation of platelet and erythrocyte on the hydrogels, thereby enhancing the hydrogel’s hemostasis effect. Preparing the hydrogels without the use of initiators or crosslinking agents is important for the safe application of the hydrogels. The good mechanical features of the hydrogel and its long-term stability resulting from electrostatic interactions and hydrogen bonds in the hydrogel network are crucial. In vivo studies in a full-thickness cutaneous infected wound model revealed the hydrogel’s capability to accelerate over 95% of wound healing within two weeks [93]. The development of hydrogels with long-term antimicrobial effects and efficient hemostasis effects is appropriate for infected wounds.

### 3.4. Nanofibers

Nanofibrous wound dressings are characterized by a highly porous structure useful for the effective permeability of oxygen, water, and nutrients [111,112]. They also display a high surface area that is important for enhanced hemostasis. They protect the wound from infections by providing excellent conformability and coverage of the wound [111,113]. Their high surface area is useful for high drug loading. Nanofibers designed for wound dressings are classified based on cladding as uni-axial, coaxial and tri-axial nanofiber [114]. The uni-axial nanofibers are prepared by the facile electrospinning technique. Recently, there are several reports of drug-loaded nanofibers via a coaxial electrospinning technique comprising a core of shell structure where the drug is loaded and released depending on the thickness of the shell, biodegradability, porosity, etc. [114] Coaxial nanofibers retain the loaded drug, minimizing high burst release effects. Coaxial nanofibers are classified into four types namely: nanofibers with different polymers in the core and sheath; biological molecules loaded in the core and the sheath in the polymer; drug-loaded nanofibers in the core and polymer in the sheath; and hybrid nanofibers loaded with more than one drug in the core or the core and sheath [115]. The nanofibers are further classified into five classes: nanofibers comprising a pristine synthetic or natural polymer; polymer blend nanofibers prepared from more than one polymer; polymer nanofibers loaded with biological molecules, e.g., growth factor, cells; polymer nanofibers loaded with drug; and hybrid nanofiber loaded with a combination of drug or a drug and biological molecule [114].

Nanofibers are prepared by several methods such as phase separation, self-assembly, electrospinning, etc. [115]. However, electrospinning is a widely reported method used to develop nanofibers due to its cost-effectiveness, simplicity, and versatility [116]. The 3D structure of nanofibers imitates the skin’s ECM and is crucial in cellular adhesion and proliferation [117]. There are several reports on the design of nanofibers for wound healing. Some of them are prepared from biopolymers such as chitosan, alginate, hyaluronic acid, gellan gum, cellulose, gelatin, keratin, pectin, and xanthan gum in combination with synthetic polymers. Most biopolymers exhibit excellent properties useful in the design of wound dressings. However, their high surface tension and viscosity make them unsuitable for electrospinning in their natural form. They are combined with known synthetic polymers, resulting in materials with interesting features.

Chitosan in combination with selected synthetic polymers has been used to prepare nanofibers for wound healing, which was reported between 2020 and 2022. Chitosan–polyethylene oxide nanofibers loaded with silver and zinc oxide nanoparticles exhibited enhanced antibacterial activity against *E. coli*, *S. aureus*, and *P. aeruginosa* with a high antioxidant effect. No cytotoxic effects of the nanofibers were visible on the fibroblast cells in vitro. The nanofibers displayed good blood compatibility, fibroblast migration, and proliferation on the wound margin in vitro. The combination of the nanoparticles produced a synergistic antibacterial effect. The nanofibers displayed good tensile strength [118]. Polyurethane–chitosan nanofibers were designed for the treatment of diabetic wounds. The nanofibers were loaded with different concentrations of linezolid. In vivo studies in streptozotocin-induced diabetic rats showed that the nanofibers comprising linezolid promoted the healing of diabetic wounds. The nanofibers displayed good mechanical features and high porosity effective for cellular attachment, differentiation, and proliferation with an increase in wound contraction [119]. Chitosan–polyethylene oxide nanofibers loaded with teicoplanin (2 and 4 *w*/*v*%) were evaluated as potential wound dressings for the treatment of infections in orthopaedic surgery. The drug release was sustained for 12 days. The antibacterial effect of the drug-loaded nanofibers on *S. aureus* was significant with no cytotoxic effect on human fibroblast. In vivo study on a rat full-thickness wound model revealed accelerated wound closure for nanofibers containing 4% teicoplanin [120].

Due to the therapeutic efficacy of silica in collagen creation and the capability of its degraded products to accelerate wound healing, it has been loaded into nanofibers for wound healing. Chitosan–polyethylene oxide–silica nanofibers were prepared by a combination of the sol-gel and electrospinning techniques. Ciprofloxacin was loaded into the nanofibers. The nanofibers were effective against *S. aureus* and *E. coli* with no cytotoxic effects against HFFF2 human foreskin and L929 mouse fibroblasts. In vivo evaluation using the dorsal cutaneous wounds of the Balb/C mice showed reduced infection and inflammation. The nanofiber accelerated skin regeneration, which was confirmed by increased fibroblast cells and angiogenesis [121]. Chitosan–polyethylene oxide nanofibers were also loaded with Manuka honey, propolis, *Calendula officinalis* infusion, insulin, and *L*-arginine [122]. The nanofibers were biodegradable with improved hemocompatibility and reduced cytotoxic effect. The nanofibers loaded with a combination of propolis and *Calendula officinalis* infusion displayed an improved hemolysis index of 2.92 ± 0.16%, non-toxic and radical scavenging effects, and increased antimicrobial effects against *S. aureus* compared to other nanofibers [122]. *Calendula officinalis* extract was loaded into chitosan–polyethylene oxide nanofibers prepared by electrospinning. The nanofiber diameter was in the range of 143–252 nm. The nanofibers’ antibacterial effects were high against Gram-positive and Gram-negative bacteria with 96% and 94% reduction, respectively. The wound dressings accelerated wound healing with 87.5 % wound closure in 14 days. Improved collagen synthesis, re-epithelialization, and remodeling of the wound dressings revealed their usefulness in the treatment of skin wounds [123]. Vancomycin was loaded into chitosan–poly ethylene oxide nanofibers via a blend-electrospinning process. In vivo wound healing of the drug-loaded nanofibers in full-thickness skin wounds of rats showed accelerated wound healing. The nanofiber containing 2.5% of the drug displayed a higher Young’s modulus and tensile strength with the fastest wound healing [124].

Chitosan–polycaprolactone nanofiber has been developed for wound dressings. Polycaprolactone is biodegradable and non-toxic, but it is hydrophobic with no interaction with native body cells [125]. It is combined with biopolymers to enhance biocompatibility. *Melilotus officinalis* (*MO*) extract was loaded into the nanofiber. The tensile strength was 11.45 ± 0.12 MPa with Young’s moduli of 31.57 ± 0.83 MPa, which is appropriate for normal skin. The nanofiber was effective against *Bacillus* and *Shigella* with no toxicity [126]. Curcumin-loaded chitosan–polycaprolactone nanofibers were prepared by electrospinning followed by electrospraying of curcumin–chitosan nanoparticles into the nanofibers. The nanofibers displayed improved antibacterial activity against MRSA and increased antioxidant activity. The nanofibers with electrospray nanoparticles induced 96.4% wound healing in MRSA-infected wounds. A complete wound healing process was observed on day 15 [127]. Quercetin and rutin were loaded into chitosan–polycaprolactone as wound dressings for burn wounds. The presence of chitosan in the scaffold improved the hydrophilicity, water absorption capacity and the specific surface area. The nanofiber loaded with quercetin exhibited superior features. The nanofibers displayed good biocompatibility, showed promising antibacterial activity, and were found to be useful for the treatment of burn wounds [128].

Hyaluronic acid has been employed in the design of nanofibers for wound dressings. It has been combined with polyvinyl alcohol and *L*-arginine [129] and in combination with polyvinyl alcohol and polyethylene oxide [130]. Hyaluronic acid-poly vinyl alcohol-L-arginine nanofibers were loaded with cellulose nanocrystals as nanofiller and L-arginine to accelerate wound healing. The cellulose nanocrystals improved the mechanical and swelling properties of the nanofibers significantly. It exhibited excellent hemocompatibility together with the significant cellular proliferation of normal human skin melanocytes. The release of arginine from the nanofibers was sustained with 90% release in 48 h. The antibacterial activity against *K. pneumonia* was significant [129]. Hyaluronic acid-poly vinyl alcohol-polyethylene oxide nanofibers were loaded with a combination of zinc oxide nanoparticles and cinnamon oil. The nanofibers’ antibacterial activity against *S. aureus* was significant. In vivo studies showed high antibacterial activity of the nanofibers in full-thickness wounds inoculated with *S. aureus* with accelerated healing [130]. Hyaluronic acid-poly ethylene oxide nanofibers were loaded with different natural-based bioactive agents such as L-arginine, propolis, Calendula officinalis infusion, and Manuka honey. The nanofiber membranes loaded with propolis and Calendula officinalis displayed outstanding cytocompatibility, antioxidant, and antimicrobial activities (against pathogen *E. coli*, *S. aureus*, and *P. aeruginosa*). The water vapour transmission rate of the nanofibers was in the range of 4634 g/m^2^.24 h–5122.6 g/m^2^.24 h, indicating their capability to prevent wound dehydration. The combination of propolis and *Calendula officinalis* induced synergistic antibacterial effects [131]. Polygalacturonic acid–hyaluronic acid nanofibers loaded with silver nanoparticles via electrospinning displayed excellent antibacterial activity and accelerated wound healing in the albino rat model with high wound epithelization and collagen deposition on day 14 after administration [132]. 

Movahedi et al. reported core–shell polyurethane–starch–hyaluronic acid nanofibers prepared via a coaxial electrospinning technique. The average fiber diameter was 428 ± 78.32 nm with an average porosity of 3.186 ± 0.401 μm. The nanofibers were non-toxic and biocompatible. Modification of nanofibers with hyaluronic acid significantly enhanced the adhesion of cells to the nanofibers in vitro, making nanofibers suitable for skin regeneration [133]. An ethanolic extract of propolis has been loaded into polyurethane–hyaluronic acid nanofibers for wound dressing. The nanofibers loaded with 1% and 2% of the extract displayed enhanced antibacterial activity against *S. aureus* and *E. coli*. The nanofibers containing a 1% extract displayed excellent biocompatibility on L929 fibroblast cells with accelerated wound healing and closure, which was visible with improved development of dermis, hair follicles, and deposition of densely packed collagen on the healed wound area [134].

Gelatin has been employed for the design of nanofibers. Combining multiple drugs to overcome multidrug-resistant pathogens was performed by loading ciprofloxacin and tetracycline hydrochloride into coaxial polycaprolactone–gelatin nanofibrous wound dressings. Ciprofloxacin was loaded into the core layer of polycaprolactone, while tetracycline hydrochloride was loaded into the gelatin shell layer. The release of ciprofloxacin was sustained for five days with over 80.71%, and the release of tetracycline hydrochloride was rapid in 12 h with over 83.51% release. The antibacterial activity of the nanofiber against *E. coli* and *S. aureus* was excellent with outstanding biocompatibility on human skin fibroblast cells [135]. Epigallocatechin-3-O-gallate was loaded into poly (L-Lactic-*co*-caprolactone)–gelatin–core-shell nanofiber membrane prepared using coaxial electrospinning technology. The nanofibers facilitated accelerated wound closure with good tissue organization and excellent hemostatic ability. The nanofibers provided suitable structural features to accelerate the healing process [136]. ZIF-8@gentamicin were loaded into polyacrylonitrile–gelatin nanofibers for wound healing. Synergistic antibacterial effects were significant with accelerated wound healing time in 16 days [137]. Hibiscus rosa–Sinensis leaves mucilage–polyvinyl alcohol–pectin nanofibers accelerated wound healing on Swiss albino mice model with rapid epithelization in 8 days. Their hemocompatibility and biodegradability nature further reveal the efficacy of combining synthetic and biopolymers [138]. Keratin has been used in combination with synthetic polymers to prepare nanofibers. The nanofibers exhibited appropriate mechanical properties such as modulus and mechanical strength similar to the natural skin. Improved cell–scaffold adhesion and the proliferation of fibroblast cells of the nanofibers revealed their capability to support wound healing. The antibacterial activity against Gram-negative and Gram-positive strains of bacteria was promising [139,140,141].

### 3.5. Nanogels

The three-dimensional hydrogel materials in the nanoscale size range are known as nanogels [142]. They are prepared from a combination of bio- and synthetic polymers. Their features such as charge, size, porosity, degradability, etc. can be tailored by modifying the chemical composition [143]. They are hydrophilic, biocompatible, and support a high drug loading capacity. They protect the loaded drugs from degradation. They are useful in extending the circulation half-lives of the loaded drug and are suitable for the combination and delivery of drug molecules [144]. Commonly reported synthesis methods of nanogels include the polymerization of monomers in a homogenous phase or heterogeneous system, physical self-assembly, crosslinking of preformed polymeric chains, and template-assisted nanofabrication. The most employed method is the physical self-assembly [145].

Fasiku et al. developed injectable nanogel to deliver a combination of antimicrobial peptide and nitric oxide. The nanogel was prepared by crosslinking divinyl sulfone with hyaluronic acid. Nitric oxide release from the nanogel was sustained for 24 h. The antibacterial activity of the nanogel was broad with antibiofilm activity. The MIC values of the nanogel were 0.39, 0.78, and 1.56 μg/mL against *P. aeruginosa*, methicillin-resistant *S. aureus*, and *E. coli*, respectively. The antibiofilm efficacy of the nanogel showed a 12.5 and 24-fold reduction in the biofilms of MRSA and *P. aeruginosa* [146]. Amato et al. designed nanogel from the combination of poly-L-lysine, hyaluronan, and berberine by the ionotropic gelation method. The release of berberine was rapid with a 50% release in 45 min in vitro followed by a sustained release of 50% in 24 h. The swelling capability of the nanogel was significant, indicating its capability to promote the release of the drug. The burst berberine release is useful for good drug concentration in situ. The wound-closure properties of the nanogels in vitro revealed a higher closure rate for the blank than the drug-loaded nanogel. The reduced wound closure of the drug-loaded nanogel is attributed to electrostatic interactions occurring between the drug, berberine and hyaluronan, resulting in a significantly reduced availability of hyaluronan needed to promote wound closure [147].

Nanogels have been designed and incorporated into hydrogels for wound dressing applications. Rusu et al. incorporated nanogels prepared from a combination maleoyl-chitosan and poly(aspartic acid) into hydrogel prepared from thiolated hyaluronic acid for wound dressings. Amoxicillin was loaded into the nanogels. The incorporation of the nanogel into the hydrogel reduced its swelling capacity and also controlled its rate of degradation, and it enhanced the rapid release of the drug at a pH of 5.4. The stability of the nanogel in physiological conditions was promoted by incorporation into the hydrogel. The formulation was biocompatible in vivo, revealing its capability to support cell proliferation and differentiation together with promoting a rapid wound healing process [148]. Zhu et al. incorporated nanogel into a hydrogel to overcome the rapid release profile that is associated with hydrogels. The nanogel prepared from a combination of lysine and di-p-nitrophenyl adipate was loaded with chlorhexidine diacetate. Incorporating the nanogel into the hydrogel prepared from a combination of aminoethyl methacrylate hyaluronic acid and methacrylated methoxy polyethylene glycol prolonged the drug release period of the drug for 240 h and also extended the antibacterial activity for 10 days. The hydrogel incorporated with the nanogel promoted rapid hemostasis and accelerated wound healing in vivo [149].

### 3.6. Films/Membranes

Film-based wound dressings are impermeable to liquid and bacteria but permeable to gas [150]. They are used for drug delivery to the wound site. They are flexible and conform easily to the skin around areas, such as the joints. They reduce pain, support moisture evaporation, inhibit external contamination, and are transparent for the inspection of the wound bed. However, their use is limited by their ability to induce pain upon removal and their inability to absorb excess exudate in high-exuding wounds [151]. Methods used for the preparation of films are solvent casting, salt leaching, spin-coating, microfluidic spinning, and 3D printing [151]. Membranes, on the other hand, display similar structures as films. However, they can absorb excess exudate and maintain a moist environment suitable for the accelerated wound healing process. Their excellent flexibility, stretchability, softness, and providing comfort are interesting features they exhibit [152,153]. Film-based wound dressings prepared from the combination of bio- and synthetic polymers have been reported by several researchers between 2019 and 2022.

**Figure 7:**
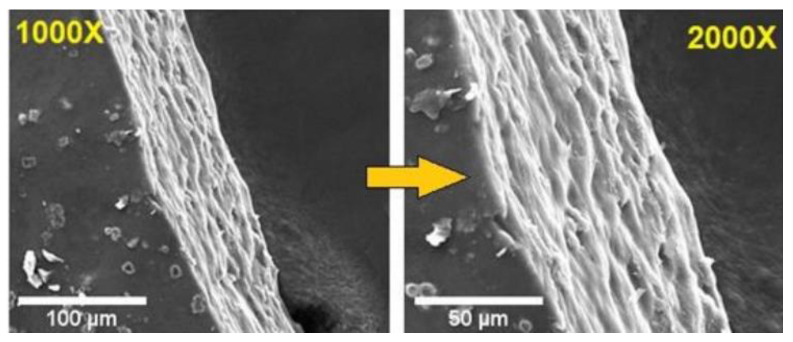
SEM images of the films loaded with amikacin [157].

Colobatiu et al. used the solvent casting method to prepare films from the combination of chitosan and poly(vinyl alcohol). The film was loaded with an alcoholic extract containing a mixture of *Arnica montana*, *Geum urbanum*, *Plantago lanceolata*, *Symphytum officinale*, *Tagetes patula*, and *Calendula officinalis*. The films provided moisture to the wound bed, thereby decreasing dehydration and accelerating wound closure. It also displayed a good proliferative effect and antioxidant activity. The films were biocompatible and induced wound contraction. In vivo studies on a streptozotocin-induced diabetic rat model showed that the films induced wound contraction with a complete re-epithelialization and a deposition of dense collagen [154]. Films have been prepared from a combination of sodium carboxymethylcellulose and hydroxypropylmethylcellulose via crosslinking using citric acid. The films were loaded with a combination of zinc oxide complex and grapefruit seed extract. The elongation (%) of the films was in the range of 34–60%, and the antioxidant activity of films was in the range of 25–79%. The release of grapefruit seed extract and zinc from films was sustained. The film’s antibacterial activity against *S. aureus* and *E. coli* was significant. The loading of the grapefruit seed extract decreased the tensile strength of the films. The film with the lowest value of tensile strength was 7.69 ± 1.14 MPa for the film loaded with 1% of the extract. The extract and zinc oxide in the films influenced the antibacterial and antioxidant activity [155]. The temperature-responsive film was prepared from pullulan and the monomer, *N*-isopropyl acrylamide, was prepared via a free radical polymerization method. Two different concentrations of silver nanoparticles (15 ppm and 30 ppm) were loaded into the films. The release of silver from the films was sustained for 48 h. The films exhibited excellent swelling properties, which is a feature useful for the absorption of wound exudates. The antibacterial activity of the films was effective against *S. aureus* and *E. coli*. Cytotoxicity evaluation on HeK293 cells showed the good biocompatibility of the films. The temperature-responsive nature of the films influenced the release of the nanoparticles when the temperature of the wound exudate was higher than normal [156]. Alruwaili et al. prepared film dressings from arabinoxylan and carboxymethylcellulose via a solvent-casting method. The films were loaded with an antibiotic, amikacin. The drug release at the wound site was rapid for 4 h followed by a sustained release for 24 h for accelerated wound healing. The films were swellable, flexible, and cytocompatible with a prolonged-drug release profile. The SEM images showed a plain surface morphology, indicating homogenous dispersion of the drug in the films (Figure 7). The features of the films showed that they are appropriate for infected and exuding wounds [157].

Elastomer film was prepared from a combination of tributyl ammonium alginate and cationic polyurethane for full-thickness wounds. The surface-modified polyurethane film with alginate displayed enhanced hydrophilicity, tensile strength (2.6 MPa), Young elastic modulus (1.8 MPa), and high stretchability. The film’s antibacterial activity was significant against *E. coli* and *S. aureus*. The films promoted rapid healing with enhanced deposition of collagen and the formation of mature blood vessels [158]. Films have been prepared by 3D printing from the combination of bio- and synthetic polymers, i.e., chitosan with poly ethylene glycol via crosslinking with genipin. In vitro mucoadhesion studies showed that the 3D-printed film adheres to the epithelial surface and is non-cytotoxic on human skin fibroblast cell lines with over 90% of cells being viable after 48 h. The film prepared in a 1:1 ratio of chitosan:polyethylene glycol exhibited high flexibility and adhered to a mucosal surface in vitro. The film’s good swelling capability is useful for the absorption of wound exudate and maintaining a moist environment. The films were not toxic but were biocompatible. The 3D-printed films are promising dressings for chronic wound healing [159]. Hybrid-based membranes have also been prepared and displayed promising features for the treatment of chronic and acute wounds.

Poly (vinyl alcohol)–carboxymethyl chitosan-based membranes prepared by a solution casting technique displayed a porous network. The membranes were effective against *E. coli* and Staphylococcus bacteria. They were also biocompatible on fibroblasts and keratinocytes in vitro, revealing their non-toxicity. The membrane’s flexibility was useful in keeping it over the wound throughout the healing process. The crosslinker and chitosan used influenced the mechanical properties of the membranes [160]. The incorporation of graphitic carbon nitride into PVA–starch membranes resulted in excellent mechanical and thermal stability, swelling capability, hydrophilicity, moisture retention capacity and water vapour transmission [161]. Incorporating titania and silver nanoparticles into the membranes imparted excellent antibacterial activity against *S. aureus* and *E. coli* with a maximum zone of inhibition of 33.25 and 37.33 mm, respectively. The membranes displayed a sustained drug release profile with complete healing in seven days in vivo in partial and full thickness excision wounds [162]. The incorporation of gentamicin into chitosan–methoxy polyethylene glycol–polycaprolactone wound dressing membranes inhibited *S. aureus* and *E. coli* growth. The membranes were thermally stable with high moisture content and swelling capability. The above-mentioned features are useful for accelerated wound healing [163].

A pH-sensitive membrane, 2-(dimethylamino)ethyl methacrylate-polyethylene oxide, was prepared in the ratio of 50:50% *v*/*v* via crosslinking with γ-irradiation (20 kGy). The antibiotics, colistin, gentamicin, and neomycin, were loaded into the membrane for improved antimicrobial activity. The drug release of colistin and neomycin was high at pH 4 and high for gentamicin at pH 7 [164]. Membranes for wound dressings have been prepared by grafting polyglutamic acid to the surface of carboxylated halloysite nanotubes followed by co-blending with collagen matrix and crosslinking with sodium alginate oxide. The elongation at the break and the tensile strength of the membranes were enhanced. The membrane was non-toxic and displayed the potential to promote cell proliferation and migration appropriate for wound healing [165].

Propolis has also been loaded into poly (vinyl alcohol)/chitosan membranes crosslinked with genipin. The loaded propolis reduced the contact angle of the membrane but enhanced the cell proliferation rate of the membrane, water uptake, and hydrophilicity. The membrane displayed good genotoxic potential and was suitable for wound healing applications [166]. A summary of hybrid-based wound dressings is shown in Table 1.

## 4. Future Perspective and Conclusions

Hybrid-based wound dressings developed from the combination of natural and synthetic polymers in the form of foam, hydrocolloid, hydrogel, nanofibers, and films/membranes have displayed promising features appropriate for accelerated wound healing. There are few reports on hybrid-based foam wound dressings. The reported hybrid-based foams are non-cytotoxic, compatible and accelerate the rate of wound closure in vivo. Their significant hemostatic and antibacterial effects due to the incorporation of bioactive agents are useful for bleeding and infected wounds. However, the limitations associated with foams that require future modification include the need for frequent changes and low exudates absorption capability. There is a need to improve the absorption capacity of foams.

In the design of hybrid-based hydrocolloid wound dressings, the presence of biopolymers plays a significant role in accelerating wound healing, exudate absorption capability, and biocompatibility of the wound dressings. However, there is limited study on the capability of hydrocolloids to support skin regeneration. There are numerous studies on the design of hybrid hydrogels with excellent features for accelerating wound healing. However, most of the studies were evaluated at the in vivo stage, and there is a need for these studies to reach the clinical trial stage.

Most of the wound dressings were loaded with synthetic antibiotics that are characterized by drug resistance and adverse side effects. The conventional approach for treating microbial infections associated with wounds involves using antibiotics. However, the persistent use of antimicrobials can induce multidrug-resistant bacteria, which poses a serious threat to human health [167]. Incorporating antibiotics into wound dressings will promote sustained and controlled drug release, thereby overcoming the risk of the development of drug-resistant microbes. There is also a pressing need to explore extensively essential oils and naturally occurring bioactive agents by incorporating them into wound dressings to further improve the biocompatibility of wound dressings. The use of chemicals such as crosslinkers in the preparation of some wound dressings is a disadvantage due to their capability to release toxic by-products that can induce adverse side effects [168,169]. Furthermore, the optimization of the large-scale production of some of these wound dressings is challenging [168].

## Figures and Tables

**Table 1:** A summary of hybrid-polymer-based wound dressings.

Synthetic Polymer	Biopolymer/Semisynthetic Polymers	Bioactive Agents	Therapeutic Outcomes	References
Foam Wound Dressings				
Polyurethane	Chitosan, sodium alginate, and hydroxypropyl methylcellulose	Silver nanoparticles and asiaticoside	Non-cytotoxic, compatible and accelerated wound closure, and the formation of granulation tissue.	[69]
Polyvinyl alcohol	Sodium carboxymethylcellulose	Stearyl trimethyl ammonium chloride	An excellent absorption capacity of excess exudate, good inhibition effect against *E. coli* and *S. aureus* with good hemostatic capability.	[70]
Hydrocolloids				
Styrene–isoprene–styrene	Sodium carboxymethyl cellulose	Silk fibroin nanoparticles	Non-toxic, supports the regeneration of the dermis layer, decreased the burn wound size and increased the density of collagen fibers in vivo.	[77]
Styrene–isoprene–styrene copolymer and petroleum hydrocarbon resin	Sodium alginate	*Centella Asiatica*	Biocompatible, induced accelerated collagen deposition, regeneration of dermis, mature epidermis, and hair follicle in vivo	[78]
Hydrogel				
Poly (vinyl alcohol)	Sodium alginate	5-hydroxymethylfurfural and silver nanoparticles	Induced proliferation and migration of human skin fibroblasts and collagen production. Good compatibility and accelerated wound healing in vivo by increasing collagen production, enhancing angiogenesis/vascularization, inducing re-epithelialization, and reducing inflammation.	[91]
Poly(*N*-isopropyl acrylamide)	Cellulose nanocrystals	Metronidazole	Slow and sustained drug release.	[92]
Poly (N-vinyl-2-pyrrolidone) and poly acrylic acid	Chitosan	Silver sulfadiazine	Controlled drug release	[93]
Polyacrylamide	Chitosan	-	Promoted collagen deposition, induced skin adnexal regeneration, and accelerated wound healing.	[94]
Polyvinyl (alcohol)	Chitosan	Nano zinc oxide	A significant antibacterial activity against *E. coli* and *S. aureus*, non-toxic, biocompatible, and effective for treating infected and exuding wounds.	[95]
Benzaldehyde-terminated polyethylene glycol	Chitosan	Vascular endothelial growth factor	Excellent tissue adhesion, hemostasis, and blood cell coagulation effects.	[96]
Polyethylene glycol	Chitosan	Silver nanoparticles	The high absorption capacity of the exudates and good antimicrobial and antioxidant properties in vitro. Improved wound healing in vivo in diabetic rabbit models.	[97]
Carbopol	Gelatin	Quercetin	Accelerated wound healing with a significant wound closure time in vivo.	[98]
-	Sodium alginate, carboxymethyl chitosan	-	Promoted wound healing in vivo in a full-thickness wound model. Facilitated re-epithelialization together with vascularization and skin regeneration.	[99]
Poly(ethylene glycol)-	Chitosan	-	Covered irregularly shaped wounds with good hemostatic effect in vivo.	[100]
Poly(vinyl alcohol)	Sodium alginate	Minocycline	Hemocompatible, non-hemolytic, and antibacterial effects in vivo against methicillin-resistant *S. aureus* (MRSA)-induced murine burn wound model.	[101]
Arginine-based poly(ester urea urethane)	Glycidyl methacrylate-modified chitosan	-	Non-cytotoxic effect and excellent antibacterial activity (eliminated 91.81% of *E. coli* and 85.59% of *S. aureus*).	[102]
Polyhexamethylene biguanide	Hyaluronic acid	Salidroside	Effective against *E. coli* (97.85%) and *S. aureus* (98.56%). In vivo studies using diabetic rat models revealed that the hydrogel promoted the high formation of granulation tissue together with the formation of subcutaneous capillaries and high collagen deposition.	[103]
Arginine–poly(ester amide)	Hyaluronic acid	-	Induced keratinocytes and accelerated re-epithelialization with a uniform density of collagen deposition in vivo.	[104]
Poly(vinyl alcohol)	Hydroxypropyl cellulose	Bovine serum albumin, glutathione, and neomycin trisulfate	Controlled drug release	[105]
Polyvinyl alcohol	Sodium alginate	Polycaprolactone microspheres loaded with fibroblast growth factor (bFGF)	A sustained release of bFGF. Accelerated wound closure, cell-induced tissue regeneration and wound healing in vivo in a burn-wound rat model. Effective against *S. aureus* and *E. coli*.	[106]
Polyvinyl pyrrolidone	Keratin	Lavender oil	Controlled and tailored drug release profiles.	[107]
Polyvinyl alcohol	Sodium alginate	Bis-quaternary triphenyl-phosphonium salt	Prolonged antibacterial activity and good hemostasis effect with accelerated wound healing.	[110]
Nanofibers				
Polyethylene oxide	Chitosan	Silver and zinc oxide nanoparticles	Exhibited enhanced antibacterial activity against *E. coli*, *S. aureus*, and *P. aeruginosa* with a high antioxidant effect. Good blood compatibility with good fibroblast migration and proliferation on the wound margin in vitro.	[118]
Polyurethane	Chitosan	Linezolid	Promoted healing in streptozotocin-induced diabetic rats in vivo.	[119]
Polyethylene oxide	Chitosan	Teicoplanin	Sustained drug release with antibacterial effect against *S. aureus*. Non-cytotoxic effect and accelerated healing on a rat full-thickness wound model.	[120]
Polyethylene oxide	Chitosan	Ciprofloxacin	Effective against *S. aureus* and *E. coli* with no cytotoxic effect on HFFF2 human foreskin and L929 mouse fibroblasts. Reduced infection and inflammation in vivo in dorsal cutaneous wounds of the Balb/C mice.	[121]
Polyethylene oxide	Chitosan	Manuka honey, propolis, *Calendula officinalis* infusion, insulin, and L-arginine	Biodegradable with improved hemocompatibility and reduced cytotoxic effect. Significant radical scavenging effects and increased antimicrobial effects against *S. aureus*.	[122]
Polyethylene oxide	Chitosan	*Calendula officinalis*	High antibacterial effects against Gram-positive and Gram-negative bacteria with 96% and 94% reduction, respectively. Accelerated wound healing with 87.5% wound closure in 14 days. Improved collagen synthesis, re-epithelization and remodeling.	[123]
Poly(ethylene oxide)	Chitosan	Vancomycin	Accelerated wound healing in full-thickness wound models in vivo.	[124]
Polycaprolactone	Chitosan	-	Biodegradable and non-toxic.	[125]
Polycaprolactone	Chitosan	*Melilotus officinalis*	Effective against *Bacillus* and *Shigella* with no toxicity.	[126]
Polycaprolactone	Chitosan	*Curcumin*	Improved antibacterial activity against MRSA and increased antioxidant activity. Induced 96.4% wound healing in MRSA-infected wounds.	[127]
Polycaprolactone	Chitosan	Quercetin and rutin	Improved the hydrophilicity, water absorption capacity and the specific surface area. Good biocompatibility and antibacterial activity.	[128]
Polyvinyl alcohol	Hyaluronic acid	Cellulose nanocrystals, arginine	Accelerated wound healing. Excellent hemocompatibility and antibacterial activity against *K. pneumonia*.	[129]
Polyethylene oxide	Hyaluronic acid	Zinc oxide nanoparticles and cinnamon oil	High antibacterial activity in full-thickness wounds inoculated with *S. aureus* with accelerated healing.	[130]
Poly ethylene oxide	Hyaluronic acid	L-arginine, propolis, Calendula officinalis infusion, and Manuka honey	Outstanding cytocompatibility, antioxidant, and antimicrobial activities (against pathogen *E. coli*, *S. aureus*, and *P. aeruginosa*).	[131]
Polygalacturonic acid	Hyaluronic acid	Silver nanoparticles	Excellent antibacterial activity and accelerated wound healing on the albino rat model with high wound epithelization and collagen deposition	[132]
Polyurethane	Starch and hyaluronic acid	-	Non-toxic and biocompatible.	[133]
Polyurethane-	Hyaluronic acid	Ethanolic extract of propolis	Enhanced antibacterial activity against *S. aureus* and *E. coli*. Excellent biocompatibility on L929 fibroblast cells with accelerated wound healing and closure with improved development of dermis, hair follicles, and deposition of densely packed collagen on the healed wound area.	[134]
Polycaprolactone	Gelatin	Ciprofloxacin and tetracycline hydrochloride	Sustained drug release with excellent antibacterial activity against *E. coli* and *S. aureus* with biocompatibility on human skin fibroblast cells.	[135]
Poly (L-Lactic-*co*-caprolactone)	Gelatin	Epigallocatechin-3-O-gallate	Facilitated accelerated wound closure with good tissue organization and excellent hemostatic ability.	[136]
Polyacrylonitrile	Gelatin	ZIF-8@gentamicin	Synergistic antibacterial effects with accelerated wound healing time.	[137]
Polyvinyl alcohol	Pectin	Hibiscus rosa–Sinensis leaves	Accelerated wound healing on Swiss albino mice model with rapid epithelization in 8 days.	[138]
Poly (vinyl alcohol), poly (Ɛ-caprolactone)	Keratin	-	Improved cell-scaffold adhesion and proliferation of fibroblast cells of the nanofibers. Good antibacterial activity against Gram-negative and Gram-positive strains of bacteria.	[139,140,141]
Nanogels				
Divinyl sulfone	Hyaluronic acid	S-Nitroso-N-acetyl-DL-penicillamine	Significant antibacterial activity against *P. aeruginosa*, methicillin-resistant *S. aureus*, and *E. coli*, respectively.	[146]
Poly-L-lysine	Hyaluronan	Berberine	Accelerated wound closure in vivo.	[147]
Poly(aspartic acid)	Maleoyl–chitosan	Amoxicillin	Good stability in physiological conditions and biocompatible in vivo	[148]
Methacrylated methoxy polyethylene glycol	Aminoethyl methacrylate hyaluronic acid	Chlorhexidine diacetate	Prolonged drug release and extended antibacterial activity. Promoted rapid hemostasis and accelerated wound healing in vivo.	[149]
Membrane/Films				
Poly(vinyl alcohol)	Chitosan	An alcoholic extract containing a mixture of *Arnica montana*, *Geum urbanum*, *Plantago lanceolata*, *Symphytum officinale*, *Tagetes patula* and *Calendula officinalis*	Good proliferative effect and antioxidant activity, biocompatible and induced wound contraction with a complete re-epithelialization and a deposition of dense collagen in vivo on a streptozotocin-induced diabetic rat model.	[154]
	Sodium carboxymethylcellulose and hydroxypropylmethylcellulose	Zinc oxide complex and grapefruit seed extract	The release of grapefruit seed extract and zinc from the films was sustained. Good antibacterial activity against *S. aureus* and *E. coli* was significant.	[155]
*N*-isopropyl acrylamide	Pullulan	Silver nanoparticles	The antibacterial activity of the films was effective against *S. aureus* and *E. coli*. Good biocompatibility on HeK293 cells and temperature-responsive nature.	[156]
-	Carboxymethylcellulose and arabinoxylan	Amikacin	Initial rapid drug release followed by a sustained release.	[157]
Polyurethane	tributyl ammonium alginate	-	Good antibacterial activity against *E. coli* and *S. aureus*. Promoted rapid healing with enhanced deposition of collagen and the formation of matured blood vessels.	[158]
Polyethylene glycol	Chitosan	-	Non-cytotoxic on human skin fibroblast cell lines with over 90% of the cells being viable. High flexibility and adherence to a mucosal surface in vitro.	[159]
Poly (vinyl alcohol)	Carboxymethyl chitosan	-	Effective against *E. coli* and Staphylococcus bacteria. They were also biocompatible with fibroblasts and keratinocytes in vitro.	[160]
Poly (vinyl alcohol)	Starch	Carbon nitride	Excellent mechanical and thermal stability, swelling capability, hydrophilicity, moisture retention capacity, and water vapor transmission.	[161]
Poly (vinyl alcohol)	Starch	Titania and silver nanoparticles	Excellent antibacterial activity against *S. aureus* and *E. coli* with a maximum zone of inhibition of 33.25 and 37.33 mm, respectively. Complete healing in seven days in vivo in partial and full thickness excision wounds.	[162]
Methoxy polyethylene glycol and polycaprolactone	Chitosan	Gentamicin	Inhibited *S. aureus* and *E. coli* growth. Thermally stable with high moisture content and swelling capability.	[163]
2-(dimethylamino)ethyl methacrylate-polyethylene oxide		Colistin, gentamicin, and neomycin	The drug release of colistin and neomycin was high at pH 4 and high for gentamicin at pH 7.	[164]
Polyglutamic acid	Sodium Alginate	-	Increased elongation at the break, tensile strength, and biocompatibility.	[165]
Poly (vinyl alcohol)	Chitosan	Propolis	Improved cell proliferation rate, water uptake and hydrophilicity. The good genotoxic potential is suitable for wound healing applications.	[166]
